# Moderate hyperosmolar hyponatremia caused by excessive off-label use of icodextrin during peritoneal dialysis 

**DOI:** 10.5414/CNCS110854

**Published:** 2023-04-12

**Authors:** Harshad Chaudhari, Smita Mahendrakar, Apokbo Akporotu, Michael Yudd

**Affiliations:** 1 Department of Medicine, Rutgers New Jersey Medical School, Newark, and; 2 Department of Medicine, Department of Veterans Affairs Medical Center, East Orange, NJ, USA

**Keywords:** icodextrin, hyponatremia, peritoneal dialysis, osmolal gap

## Abstract

Icodextrin use during the long dwell of a peritoneal dialysis (PD) regimen is commonly used to increase ultrafiltration. Its use may cause a mild and clinically insignificant degree of hyponatremia. We describe a patient who was admitted twice to our medical center on an atypical continuous ambulatory peritoneal dialysis (CAPD) regimen utilizing solely icodextrin with 2 exchanges (12-hour dwells). On both admissions, he had hyperosmolar hyponatremia in the 120-mmol/L range with a large osmolal gap. After icodextrin was stopped and his PD prescription was switched to dextrose solutions, both hyponatremia corrected and the osmolal gap quickly disappeared. The accumulation of osmotically active solute in extracellular fluids results in efflux of water from the cellular compartment and produces both hyponatremia and hypertonicity [1]. This tonic effect occurs most frequently with hyperglycemia, but other substances can also cause this, including mannitol, sorbitol, glycine, and maltose [1, 2]. In this report, we present a patient with end-stage renal disease (ERSD) on an atypical off-label PD regimen utilizing solely icodextrin solutions who developed hyperosmolar hyponatremia in the 120-mmol/L range, with a large osmolal gap. This appeared to be due to absorbed metabolites of icodextrin, mainly maltose.

## Patient history 

The patient is a 76-year-old nondiabetic man with end-stage renal disease (ESRD) due to diffuse vascular disease. He was admitted with dry gangrene of the right lower leg and was to undergo an above-the-knee amputation. He had a history of ischemic cardiomyopathy, aortic stenosis, atrial fibrillation, and partial colectomy. Medications included carvedilol, losartan, calcitriol, calcium carbonate, and a renal multivitamin. 

Five months earlier he started peritoneal dialysis (PD) in an outside clinic. There, his nephrologist placed him on an atypical continuous ambulatory peritoneal dialysis (CAPD) regimen consisting solely of icodextrin with 2-L exchanges, morning, and night. He reported a daily urine output of 700 mL and ultrafiltration of 1 – 2 L/day and a daily oral intake of 1.8 L. He was continued on this regimen during his hospitalization. 

Physical exam revealed a well-nourished elderly man. He was afebrile, with a heart rate of 88 beats per minut and a blood pressure of 110/72 mmHg. His weight, stable according to him, was 82 kg, and body mass index was 25.2 kg/m^2^. Skin turgor was normal, lungs were clear; on cardiac exam he had a regular rhythm with ectopy, and there was 1+ edema at both ankles. Dry gangrene of the right foot was noted. Serum sodium (Na) on admission was 126 mmol/L (Table 1). He was considered to be mildly overloaded, and the hyponatremia was initially attributed to excessive fluid intake in ESRD. He was fluid restricted. Serum Na rose to 131 mmol/L, and he underwent the amputation. For the remainder of this 8-day admission, serum Na remained 129 – 131 mmol/L while on oral fluid restriction of 1 L daily and mild normal saline infusions postoperatively. He was referred back to his dialysis center with the advice to switch his treatment to a dextrose-based CAPD regimen. 

Four weeks later, he was readmitted with weight loss and dry gangrene of the other foot. He described eating poorly, but he stated he adhered to the 1-L fluid restriction. His nephrologist kept him on the same icodextrin regimen as an outpatient for unknown reasons. This regimen was continued initially during this second hospitalization. 

He was afebrile, tachycardic and hypotensive with systolic blood pressure of 85 – 110 mmHg. Weight was 71 kg. He was alert and appeared volume depleted, with poor skin turgor and no peripheral edema. 

Abdomen was soft and non-tender. Dry gangrene of the left foot was present. PD effluent had 450 WBC/mL with 90% neutrophils. Blood and peritoneal fluid cultures were negative. He did not appear septic. He was treated for culture-negative peritonitis with good response. Urine output decreased to 200 mL/day. 

On this admission, serum [Na^+^] was 122 mmol/L. His volume status was corrected with infusions of 2 L of normal saline daily for the first 4 days, and oral fluid restriction of 1 L was continued. Despite correction of hypovolemia, serum Na remained in the 122 –128 mmol/L range along with measured hyperosmolality of 305 – 308 mosm/kg and a large osmotic gap, 27 mosm/L (Table 1). 

This elevated osmolar gap raised the suspicion for the accumulation of osmotically active oligoglucose osmoles in the serum from icodextrin metabolism causing the hyperosmolar hyponatremia. The CAPD regimen was switched to 3 daily exchanges of 1.5% solutions (patient refused 4 exchanges daily). After 1 day on this regimen, serum Na improved to 130 mmol/L, and the osmolar gap dropped to 9 mosm/L. On day 6 of this regimen, serum Na improved to 134 mmol/L, and the osmolal gap was zero. 

**Figure 1:**
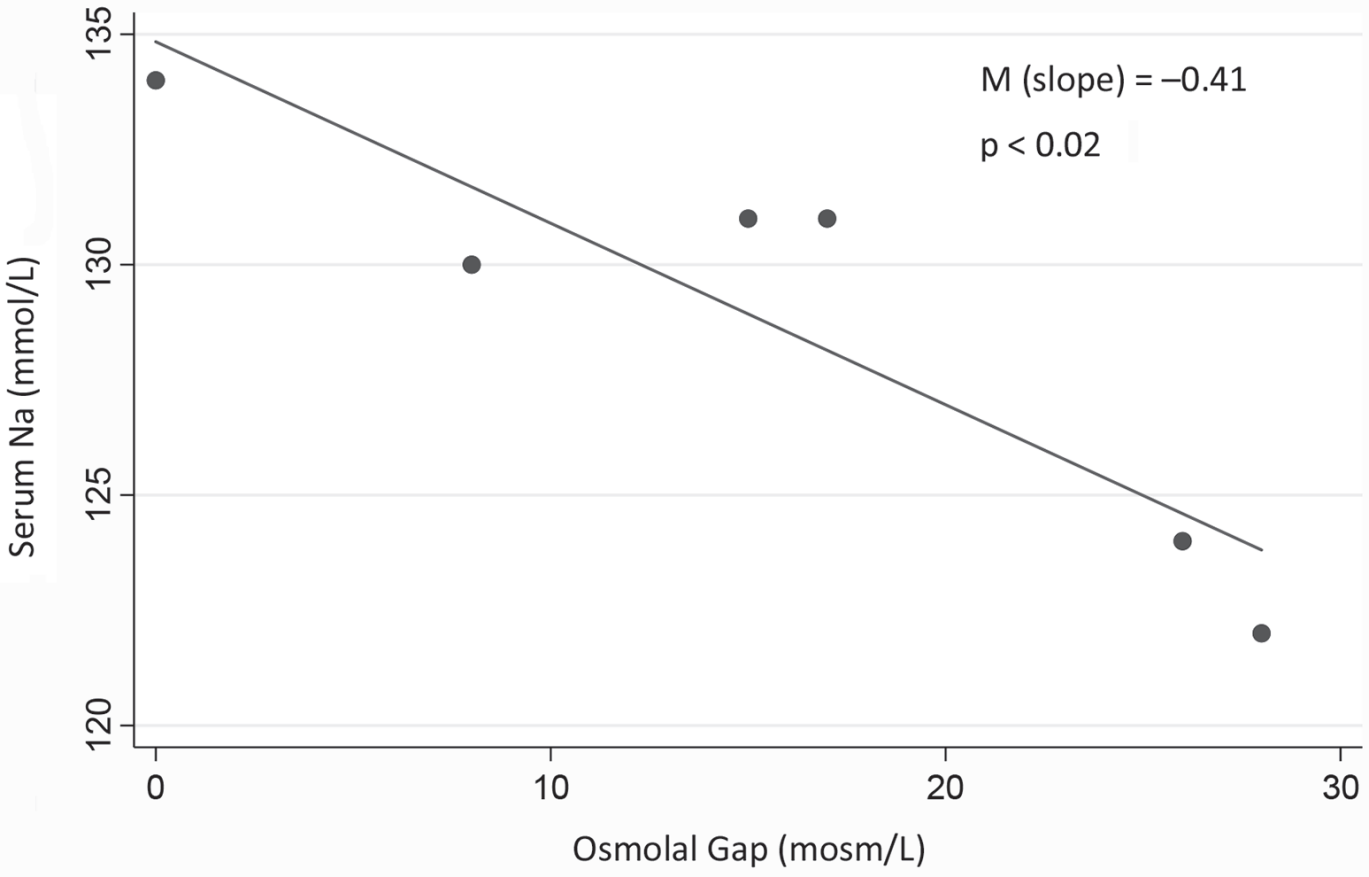
Linear regression analysis of serum sodium and the plasma osmolal gap (osmolal gap = calculated – measured plasma osmolality).

Figure 1 shows the linear regression of the osmolal gap and serum Na. The decrease of serum Na for each 1-mosm/L increase of the osmolal gap is 0.41 mmol/L. Table 2 shows the decrease in serum Na with icodextrin use reported in the literature. 

## Discussion 

Hyponatremia in patients on PD is common, with reports of incidences of 5 – 26% [1, 2, 3, 4, 9]. A useful clinical approach to hyponatremia in this population is to consider the accompanying changes in extracellular volume (ECV) status [2]. An increase in ECV or weight suggests a gain in electrolyte free water. This typically occurs with excess free water intake, and potentially excess Na intake relative to the capacity for excretion, although not to the same degree as free water excess. If there is a decrease in ECV and weight, typically with findings of volume depletion, this is due to a loss of Na and potassium salts resulting from poor nutritional intake or excess losses (e.g., gastrointestinal, peritoneal dialysate). The length of the dwell time may have some impact on the relative removal of Na and water, and subsequent serum Na levels. During short dwell times, ultrafiltration occurs mainly across “aquaporins” which remove water only. During longer dwell times, ultrafiltration shifts more across “small pores” with removal of both Na and water. 

If there is no change in the ECV status, the differential of hyponatremia includes a gain of osmotically active solutes restricted to the extracellular fluid compartment (e.g, hyperglycemia, maltose) or pseudohyponatremia (in settings of hyperlipidemia or hyperproteinemia). 

Icodextrin is a starch utilized in PD fluids for its colloidal effects to increase PD ultrafiltration. It is typically infused as 2 L of a 7.5% solution (150 gm total) for the long daily dwell, usually 8 – 16 hours. During the dwell period, icodextrin is partially metabolized to maltose and other oligoglucose polymers. These are absorbed through the lymphatics [6, 7] and remain in the extracellular fluid compartment. They raise measured plasma osmolality and the osmolal gap. The resulting osmotic gradient across the cellular membrane causes a water shift from intracellular to extracellular compartments, producing hyperosmolar hyponatremia. When icodextrin is used appropriately during the long dwells of 8 – 16 hours, the degree of hyponatremia is mild, usually a drop of 2 mmol/L or so. 

Off-label use of icodextrin solutions in our patient caused a relatively severe hyperosmolar hyponatremia, with serum Na of 122 mmol/L, measured plasma osmolality of 305 mOsm/kg and osmolal gap of 27 mosm/L. When this was recognized, and icodextrin was stopped and replaced by conventional 1.5% dextrose PD solutions, these abnormalities corrected quickly (Table 1). 

The serum Na decreased by 0.41 for every 1-mOsm/L increase in the osmolal gap in our patient (Figure 1). This factor is very close to what is found in hypertonic hyponatremia induced by glucose and maltose infusions [8, 9]. When glucose was quickly infused into normal subjects whose endogenous insulin was blocked, leading to hyperglycemia, the decrease of serum Na per 1-mmol/L increase in glucose was 0.42 mmol/L [8]. The relation was non-linear though, with smaller drops in serum Na up to serum glucose levels of 400 mg/dL, and greater decreases over 400 mg/dL. When a 10% maltose solution was infused into a patient with renal failure which caused elevated serum maltose, serum Na dropped by 0.31 for every 1-mosm/L increase in the osmolal gap [9]. From the linear regression of this maltose study, when the osmolal gap increased to 27 mmol/L, the serum Na dropped to ~ 125 mmol/L, close to our patient’s values. 

Kinetic studies of transperitoneal fluid and polyglucose transport were performed in patients treated with long dwells (16 hours) utilizing icodextrin [10]. Up to 41% of the dialysate icodextrin was metabolized over this period, and serum maltose and maltotriose levels rose to 174 mg/dL (5 mmol/L) and 155 mg/dL (3 mmol/L), respectively. These levels then decreased during the next dwell with dextrose. Serum Na decreased by 2.2 mmol/L from the start to end of the icodextrin dwell. To our knowledge, there are no kinetic data on 24-hour dwells, or on patients dialyzed with multiple continuous exchanges solely with icodextrin as our patient. We would expect the metabolites of icodextrin to increase to much higher levels in these settings. 

If we assume the baseline osmolal gap for this patient is ~ 4 mOsm/kg H_2_O (osmolal gap 0 – 9 mOsm/kg H_2_O when measured on the dextrose PD regimen), then the increase in the osmolal gap during the second admission was around 23 mOsm/kg H_2_O. Putting this in clinical perspective, if hyperglycemia had caused a similar increase in osmolality, the blood glucose would be ~ 500 mg/dL [8]. If the baseline serum Na^+^ were 134 mmol/L, the expected decrease of serum Na for this degree of hyperglycemia would be 6.4 – 8 mmol/L, diluting the Na to 126 – 128 mmol/L. 

This case highlights the following teaching points:

- The off-label use of icodextrin of excessive dwell periods approaching 24 hours (two 12-hour dwells) caused a fairly severe hyperosmolar hyponatremia with serum Na 122 mmol/L.
- If this condition is suspected, measuring the plasma osmolality and calculating the osmolal gap is necessary to recognize this condition.
- This off-label use of icodextrin should be avoided.

## Funding 

There was no funding received by any of the authors. 

## Conflict of interest 

None of the authors involved writing this paper has any conflict of interest. 

**Table 1.** Serum chemistries, osmolalities, and osmolal gap during two admissions.

Time	Sodium (mmol/L)	Glucose (mmol/L)	Urea (mmol/L)	Measured plasma OSm (mOsm/kg H_2_O)*	Calculated plasma OSm (mOsm/kg H_2_O)**	Osmolal gap (mosm/L)***
First admission						
Day 1	126	5.3	27.5		284	
Day 2	126	4.8	23.93			
Day 3	131	4.8	23.21	307	290	17
Day 9	131	5	24.64	306	292	14
Second admission						
Day 1	122	5.3	29.28			
Day 2	122	4.6	31.42	305	278	27
Day 3	124	6.1	28.21	308	282	26
Day 7	128	5.1	24.28			
Icodextrin stopped 1.5%g dextrose solution						
Day 1	130	4.3	21.78	294	285	9
Day 6	134	5.2	18.93	292	292	0
Day 8	135	5.3	18.93			

Throughout the first admission and the first week of second admission patient received solely icodextrin solutions at peritoneal dialysis. Then icodextrin solution was discontinued and replaced with 1.5% dextrose solutions. *The measured plasma osmolarity (pOsm) was performed utilizing the freezing point depression. **pOsm was calculated by the formula: pOsm = 2 (Na) + glucose (mmol/L) + urea (mmol/L). ***The osmolal gap was calculated as the difference of the osmolalities (measured pOsm – calculated pOsm).

**Table 2.** Reported decrease in serum Na+ concentration with icodextrin use in peritoneal dialysis.

Author	Year	Decrease in serum Na^+^ level (mEq/L)	Dwell time
Gradden et al. [5]	2001	Diabetics: 3.17; nondiabetics: 5.19	Long single dwell time (unspecified)
Gokal et al. [11]	2002	3.6	8-hour overnight dwell
Plum et al. [12]	2002	3 – 7	Mean long dwell time: 13.4 ± 1.2 hours
Wolfson et al. [13]	2002	2.8	Long dwell (dwell time, 8 to 16 hours)
Dimitriadis et al. [14]	2014	3.5	Not specified
Olszowska et al. [10]	2019	2.2	16-hour dwell
